# Fingerprint Recognition in Forensic Scenarios

**DOI:** 10.3390/s24020664

**Published:** 2024-01-20

**Authors:** Nuno Martins, José Silvestre Silva, Alexandre Bernardino

**Affiliations:** 1 Portuguese Military Academy, 1169-203 Lisbon, Portugal; nuno.daniel.martins@tecnico.ulisboa.pt; 2 Instituto Superior Técnico, Universidade de Lisboa, 1049-001 Lisbon, Portugal; alex@isr.tecnico.ulisboa.pt; 3 Military Academy Research Center (CINAMIL), 1169-203 Lisbon, Portugal; 4 Laboratory for Instrumentation, Biomedical Engineering and Radiation Physics, Universidade de Coimbra (LIBPhys-UC), 3000-370 Coimbra, Portugal; 5 Institute for Systems and Robotics (ISR), 1049-001 Lisbon, Portugal

**Keywords:** fingerprints, biometrics, polygons, minutiae, forensic analysis

## Abstract

Fingerprints are unique patterns used as biometric keys because they allow an individual to be unambiguously identified, making their application in the forensic field a common practice. The design of a system that can match the details of different images is still an open problem, especially when applied to large databases or, to real-time applications in forensic scenarios using mobile devices. Fingerprints collected at a crime scene are often manually processed to find those that are relevant to solving the crime. This work proposes an efficient methodology that can be applied in real time to reduce the manual work in crime scene investigations that consumes time and human resources. The proposed methodology includes four steps: (i) image pre-processing using oriented Gabor filters; (ii) the extraction of minutiae using a variant of the Crossing Numbers method which include a novel ROI definition through convex hull and erosion followed by replacing two or more very close minutiae with an average minutiae; (iii) the creation of a model that represents each minutia through the characteristics of a set of polygons including neighboring minutiae; (iv) the individual search of a match for each minutia in different images using metrics on the absolute and relative errors. While in the literature most methodologies look to validate the entire fingerprint model, connecting the minutiae or using minutiae triplets, we validate each minutia individually using n-vertex polygons whose vertices are neighbor minutiae that surround the reference. Our method also reveals robustness against false minutiae since several polygons are used to represent the same minutia, there is a possibility that even if there are false minutia, the true polygon is present and identified; in addition, our method is immune to rotations and translations. The results show that the proposed methodology can be applied in real time in standard hardware implementation, with images of arbitrary orientations.

## 1. Introduction

Fingerprints accompany all human beings from birth. They are a biometric key composed of unique patterns found on the distal phalanges of the fingers, distinct for each individual, and can be used for various purposes. Due to their unequivocal and invariant properties, fingerprints have gained importance in the field of forensic analysis, becoming a relative alternative to other traditional authentication methods [1,2,3]. Currently, there is a growing number of applications using fingerprint recognition systems, such as for accessing mobile phones, monitoring employee presence in a company and, in forensic investigations, to achieve the unequivocal identification of an individual. Technological advances in fingerprint processing enable capture, storage, and comparison methods to be more financially accessible today, allowing a significant portion of the population to use these technologies [4,5,6].

Fingerprints can be used to determine if two images are of the same finger, thereby identifying the individual to whom it belongs. Identification of fingerprints collected in a crime scenario is typically done manually, consuming a lot of time, which may be relevant and indispensable to solve the crime. Considering that many fingerprints are collected, a lot of time is spent on comparisons that a computer system can perform in seconds, and it must also be considered that the images collected in the field are sometimes of poor quality [7].

The main problem for which a solution is sought corresponds to the identification of a fingerprint based on comparison with others, with the aim of identifying those that correspond and belong to the same individual. It is essential to find a method that can be used to make reliable comparisons, generate precise outcomes, and requiring minimal computing power. This way, fingerprint comparison can be performed in the field in real time, with the help of a mobile computing device.

In this paper, the main contributions are:

- A methodology is proposed that can accurately and efficiently to compare two fingerprints and classify them as belonging to the same person or different individuals. The proposed method can be used on portable devices during field work providing real-time screening of collected fingerprints. The proposed methodology also looks forward to validating each minutia individually while in the literature most methodologies look up to validate the entire fingerprint model.
- A new process to validate extracted minutiae is proposed using the convex hull of the set of minutiae to create a region of valid minutiae, and an individual minutia validation is proposed by employing n-side polygons instead of triangulations, which is the approach most common in the literature.

This paper is organized into five sections. The first provides a brief introduction to the work, describing the problem and motivation for carrying out the work. Section 2 details a comparison of the databases used in the reviewed articles and a study of the state-of-the-art on fingerprint comparison methods which encompasses the gaps found. Section 3 defines the proposed methodology to achieve the objectives of the work. Section 4 contains the results of applying the proposed methodology in different databases, initially tuning the algorithm in one database, and then validating it in different ones. Section 5 is where the conclusions of the work are drawn, namely about the methodology applied and the produced results, the achieved objectives, and the proposals for future work.

## 2. Background

This section covers concepts relevant to the work to be developed, namely about fingerprint features and minutiae extraction.

### 2.1. Fingerprint Features

**Figure 1:**
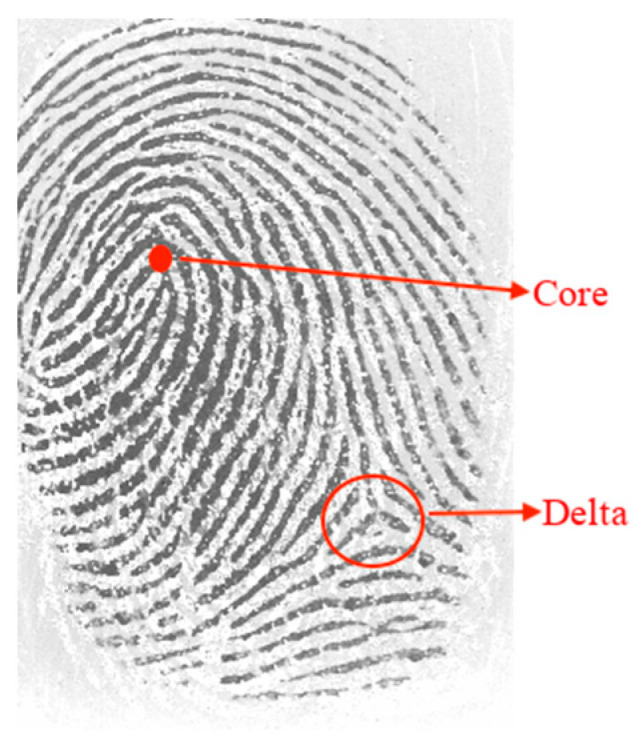
Global features: core and delta.

The features extracted from the fingerprints are organized hierarchically into global and local [8]. Global features are singular points in the fingerprint, namely the core and deltas. The core represents the point of convergence of the pattern. Deltas are points where the ridges diverge, and a point is formed that resembles the delta symbol (Figure 1). 

**Figure 2:**
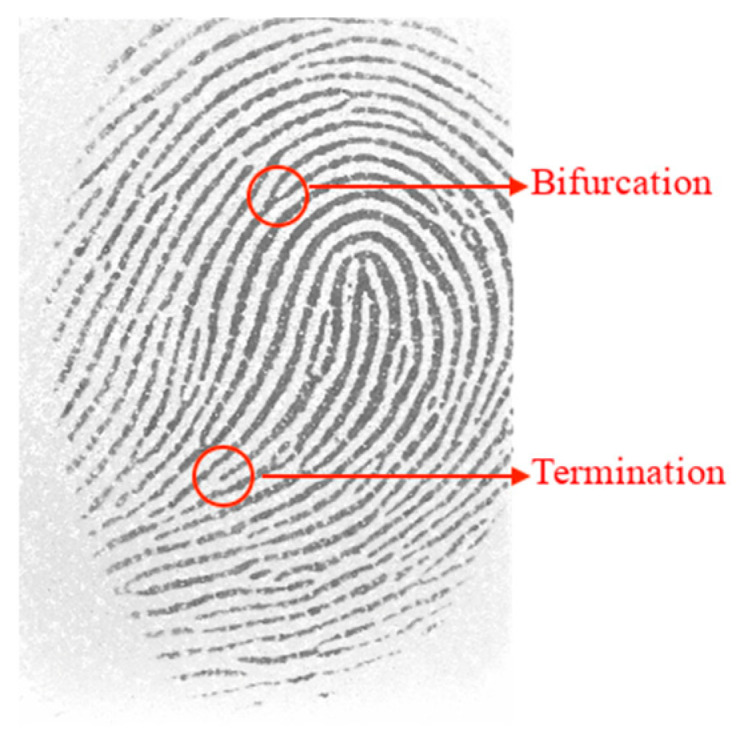
Local features: termination and bifurcation.

The local features of a fingerprint (see Figure 2) are minutiae and refer to the points at which the ridges join or end (bifurcations and terminations, respectively) and are of high relevance as they are used by most fingerprint matching algorithms (sometimes associated with global features). In a fingerprint image, depending on the quality and size, typically 10 to 200 minutiae can be found, and a good quality image should allow the identification of at least 50 to 100 minutiae. Each minutia is associated with a position and orientation, and its distribution is not uniform.

### 2.2. Crossing Numbers Method

The Crossing Numbers method is widely used to extract minutiae from a fingerprint image [9]. The termination and bifurcation extraction is reached through the analysis of the neighborhood of each pixel in the skeletonized image of the fingerprint using a 3 × 3 window centered in the reference pixel p:

$$\left[\begin{array}{c} {\;p}_{1}\;\\ {\;p}_{8}\;\\ {\;p}_{7}\; \end{array}\begin{array}{c} {\;p}_{2}\;\\ p\\ {\;p}_{6}\; \end{array}\begin{array}{c} {\;p}_{3}\;\\ {\;p}_{4}\;\\ {\;p}_{5}\; \end{array}\right]$$

The crossing number for the pixel p is computed through the difference between adjacent pixel values:

(1) $$CN=\frac{1}{2}\;\sum_{i=1}^{8}\left|P_{i}-P_{i+1}\right|\;,\;\;\;\;\;\;\;\;\;\;\;\;P_{9}=P_{1}$$

Each pixel is then labeled accordingly with its *CN* value following Table 1, and a first set of local feature points is found.

## 3. Related Work

In this section, selected works relevant to the topic are reviewed. The section is divided into subsections, analyzing the databases most mentioned in the reviewed works, the evaluation metrics of the proposed algorithms and the proposed methods, organized according to their main characteristic (based on minutiae, deep learning, and image texture). 

### 3.1. Databases

In this section, a systematic study of the databases used in the reviewed articles is carried out. An analysis is made to the frequency of each database usage, its images, and their public availability. 

**Figure 3:**
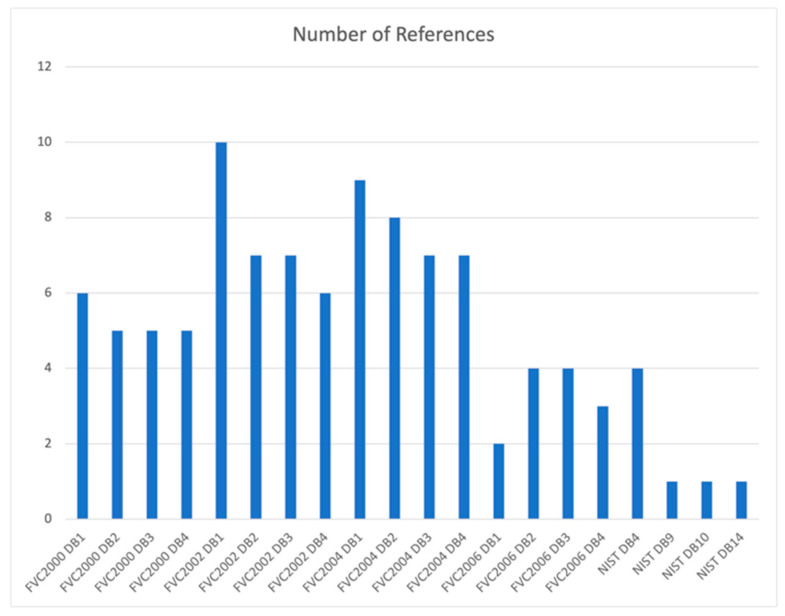
Databases used in the reviewed articles.

In Figure 3, it is observed that the databases that are most commonly used are generally those made available in the Fingerprint Verification Competition (FVC) [10]. Some authors used private databases, built for the purpose of the study, which are not included in the presented analysis. In total, there are references to 20 databases, four of which have already been discontinued, and the rest are publicly accessible, except for the FVC2006 set, which is only available for academic purposes. The FVC2000 DB4, FVC2002 DB4, FVC2004 DB4 and FVC2006 DB4 databases are composed of fingerprint images created using the synthetic fingerprint generation software SFinGe (FVC2000—not mentioned, FVC2002—SFinGe v2.51, FVC2004—SFinGe v3.0, FVC2006—SFinGe v3.0). The databases are not recent, but in the case of fingerprints, this situation is acceptable since despite being older, the images maintain their usefulness. The FVC2000, FVC2002, FVC2004, and FVC2006 datasets are the most used, being composed of images acquired through different sensors, presenting different challenges to fingerprint recognition and matching systems.

#### 3.1.1. FVC2000

Table 2 summarizes the FVC2000 [11] database composed of four datasets, each with eight images of 110 fingers up to a total of 880 images.

Different sensors represent various challenges for the algorithms.

**Figure 4:**
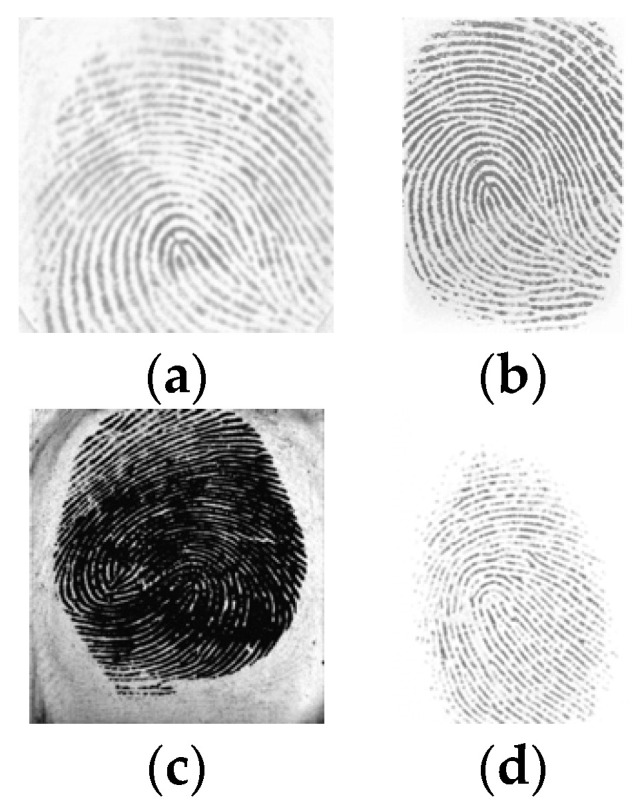
FVC2000: (**a**) DB1, (**b**) DB2, (**c**) DB3, (**d**) DB4.

Figure 4 shows some examples of FVC2000 images, one for each dataset.

#### 3.1.2. FVC2002

Table 3 summarizes the FVC2002 [12] database: 4 datasets with 8 images of 110 fingers each, up to a total of 880 images.

**Figure 5:**
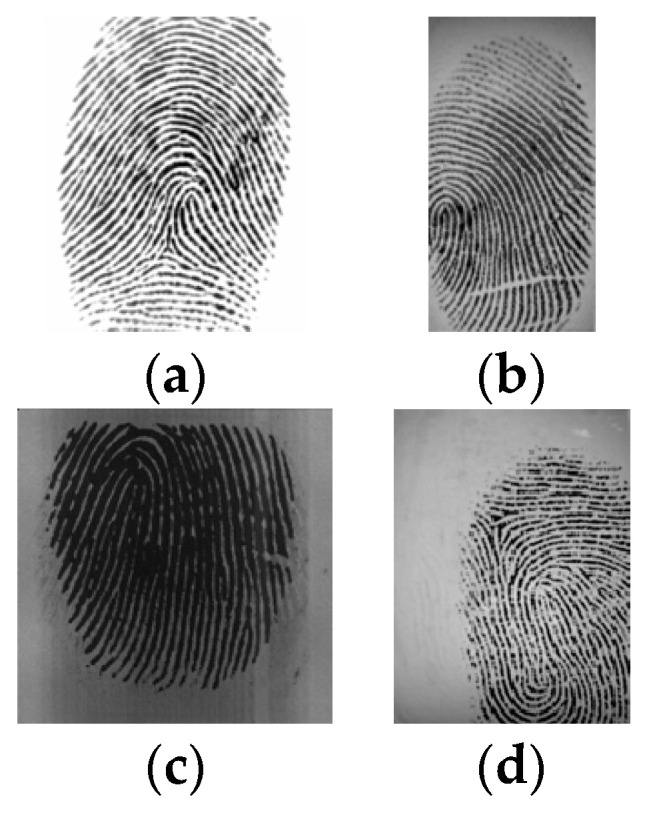
FVC2002: (**a**) DB1 (**b**) DB2 (**c**) DB3 (**d**) DB4.

Figure 5 shows some examples of FVC2002 images, one for each dataset.

#### 3.1.3. FVC2004

Table 4 summarizes the FVC2004 [13] database: 4 datasets and 8 images of 110 fingers each, up to a total of 880 images. This database contains images to which purposeful difficulties have been added to make the competition more challenging, such as rotating the angle of the image, asking volunteers to press harder on the sensor (introducing distortions), and collecting images from moistened fingers.

**Figure 6:**
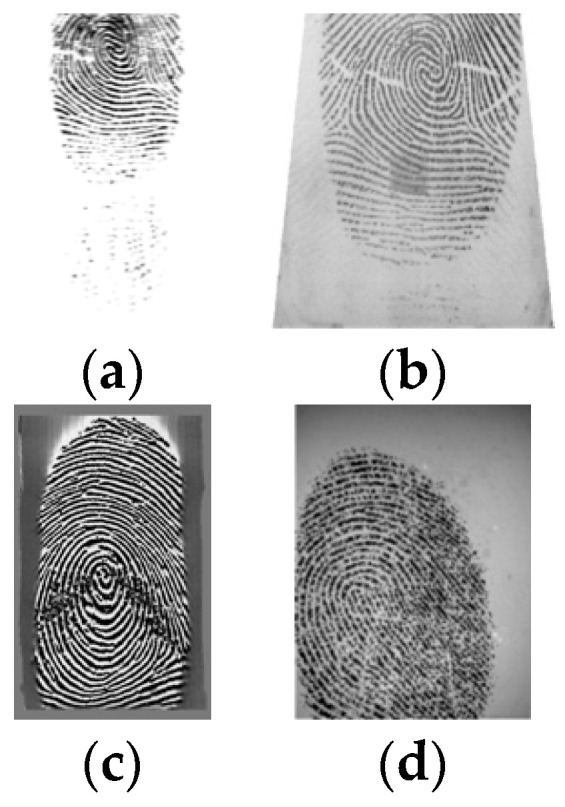
FVC2004: (**a**) DB1 (**b**) DB2 (**c**) DB3 (**d**) DB4.

Figure 6 shows some examples of FVC2004 images, one for each dataset.

#### 3.1.4. FVC2006

Table 5 summarizes the FVC2006 [14] database which has four datasets, each composed of 12 images of 150 fingers, up to a total of 1800 images per dataset.

The availability of the FVC2006 database is exclusively for academic purposes, and no response was received from the University of Bologna to provide access to the images.

### 3.2. Evaluation Metrics

In this work, the most common metrics are used to evaluate the performance of fingerprint matching systems [15]. First, it is necessary to define the concepts that allow computing of those metrics. Considering the matching of two images, we define:

- TP—True Positive: should match and match.
- FP—False Positive: should not match and match.
- TN—True Negative: should not match and do not match.
- FN—False Negative: should not match and match.

The following metrics are computed:

Recall: is the metric used to evaluate the model’s ability to correctly identify all relevant positive cases in a dataset, that is, it is the proportion of true positives (positives that are correctly identified) compared to the total positive cases existing in the dataset. This metric is useful in situations where it is crucial to identify all positive cases, even if it means incorrectly classifying some negative cases as positive.

(2) $$Recall=\frac{TP}{TP+TN}$$

Precision: evaluates the proportion of positive identifications that were obtained correctly. This metric is useful in situations where the cost of a false positive case is high.

(3) $$Precision=\frac{TP}{TP+FP}$$

F1: recall and precision have a trade-off relationship, that is, increasing one may decrease the other. The F1 metric is used to combine both into a single metric that balances the previous ones.

(4) $$F1=2\times \frac{Precision\times Recall}{Precision+Recall}$$

False Match Rate (FMR): evaluates the probability that the system will identify two fingerprints that do not match as matching.

(5) $$FMR=\frac{FP}{FP+TN}$$

False Non-Match Rate (FNMR): evaluates the probability that the system will identify two fingerprints that match as non-matching.

(6) $$FNMR=\frac{FN}{FN+TP}$$

Equal Error Rate (EER): measures the balance between FMR and FNMR. Considering different decision thresholds (values at which the system defines the images as corresponding or not), the EER is defined as the point at which these metrics are equal, that is, when the system makes a wrong decision with the same frequency (false positive and false negative).

All the mentioned metrics vary in the range $\left[0,\;1\right]$. System performance will be optimal when the recall, precision, and F1 metrics have a value of one, and FMR, FNMR, and EER have a value of zero. Typically, the values might also be represented as a percentage.

### 3.3. Minutiae Extraction and Matching

In this section, recent articles on minutiae extraction and matching are reviewed. A division is made into subsections that group the mentioned articles, considering methods based on minutiae, based on deep learning, and based on image texture.

#### 3.3.1. Minutiae Based Methods

Different methods are proposed based on the minutiae, especially in their geometric arrangement and the creation of a geometric model that represents the fingerprint.

Trivedi et al. [16] propose the creation of a non-invertible model to represent fingerprints. Images are pre-processed through normalization, binarization, skeletonization, and the application of Gabor filters. Minutiae triangles are formed, and those that cover a small area are discarded. Each validated triangle is described through feature vectors formed by the internal angles and the type of minutia that compose the triangulation. In the matching phase, for each triangle in one image, the triangle in the other image whose descriptor achieves the smallest Euclidean distance is selected. If the distance is below a threshold, the match is validated. The FVC2000, FVC2004 and FVC2006 databases were used, achieving EER of 5.57%, 2.56% and 0.48%, respectively.

Mohamed-Abdul-Cader et al. [17] propose the use of Delaunay triangulations. Images are pre-processed through histogram equalization, binarization, Gabor filters, and skeletonization. Delaunay triangulation is used to form triangles and for each a descriptor is built with the internal angles, the type of minutiae, and the minutiae orientations. Absolute and relative distances are used to select the best triangle match, and a matching score is computed using the number of corresponding minutiae (unique minutiae in the set of validated triangles) over the total minutiae in the model. A set of 158 images was selected from the FVC2002 DB1 database, achieving an EER of 6.68%.

Ghaddab et al. [18] propose the use of an expanded Delaunay triangulation, considering all possible triangulations if each minutia was removed from the set, increasing the robustness of the algorithm against false minutiae and increasing the complexity and computational effort. Each triangle is then associated with a feature vector that includes the elongation of the Steiner ellipse, the cosine of the largest angle, the perimeter, and the angle of rotation required to superimpose one vertex on the others. Considering two triangles that could possibly match, a threshold is applied to the distance between each feature in the feature vectors to validate the match. The FVC2000, FVC2002, and FVC2004 databases were used achieving EER of 2.25%, 1.62%, and 3.99%, respectively.

Surajkanta and Pal [19] also use Delaunay triangulation. Images are pre-processed by applying normalization, binarization, Gabor filters, and skeletonization. The feature vectors are built using the internal angles of each triangle, the type of minutiae, and their orientation. However, in contrast to other authors, the orientation of the minutiae is computed in relation to the core point of the fingerprint. In the FVC2000 database, an average EER of 6.68% was achieved across all four datasets.

#### 3.3.2. Deep Learning Based Methods

Liu et al. [1] propose the use of a Convolutional Neural Network (CNN) based on the VGG-16 network. The images are not pre-processed, but alignment is performed, and transformations are subsequently applied to generalize the model. The number of convolutional layers and the cost function were adjusted for optimal performance. The proposed CNN achieved a precision of 98,42% in the FVC2000 database, compared to 97.85% produced by the VGG-16 CNN.

Li [15] proposes the use of a CNN to extract features and match noisy fingerprints. The proposed method achieved FMR 1.54% and FNMR 1.46% in the NIST DB4 database and was compared with a traditional method (based on coordinates and orientation of minutiae) that produced 28.82% and 28.78%, respectively.

Gorgel and Eksi [3] suggest pre-processing by applying normalization, binarization, and the Gabor Wavelet Transform (GWT) and then using a CNN to classify the images. The FVC2006 images were used, some images were replicated, and small variations were added to generalize the CNN. The proposed method achieved a precision of 91.50% and, using the same model without using the GWT 86.27% were achieved.

Engelsma et al. [20] propose a network composed of three sub-networks for image alignment, texture-based feature extraction, and minutiae extraction, resulting in an image representation whose dimensionality is reduced through a fully connected layer. The Euclidean distance between descriptors of fixed size is computed and, subsequently, the cosine similarity is computed, applying a threshold value to the result to validate or reject the correspondence. In the FVC2004 DB1 database, rank-1 accuracy of 99.5% and rank-100 accuracy of 100% were achieved.

Tang et al. [21] propose the use of a deep convolutional network to enhance the and extract the orientation field and minutiae map from latent fingerprints. The traditional methods are transformed to convolutional kernels and integrated as a shallow network with fixed weights and then the representation ability of deep learning is used to create a minutiae map. The model was trained on 8000 pairs of matched rolled fingerprints and experiments were conducted in NIST SD27 and FVC2004 databases outperforming other compared methods.

Cao and Jain [22] propose the use of a CNN for image enhancement and for ridge flow estimation and minutiae descriptor extraction. Complementary templates (minutiae and texture) are considered to represent the latent fingerprints. Experimental results on the NIST SD27 database achieved rank-1 identification accuracy of 64.7% while 75.3% were achieved in WVU latent database.

He et al. [23] propose a partial fingerprint verification network based on spatial transformer network and the local self-attention mechanism. The model was trained end-to-end and learned multi-level fingerprint features automatically. The network considers an image pair, performs its alignment and the matching, using affine transformations and fusing features. On the AES3400 dataset, EER of 8.6% was achieved and 3.2% in FVC2006 database.

Cui et al. [24] propose an end-to-end network to directly output pixel-wise displacement field between two fingerprints, including a siamese network for feature embedding, and a following encoder-decoder network for regressing displacement field. The algorithm consists of two steps: minutiae-based coarse registration and CNN-based fine registration. Compared to other image correlation methods, this method achieved better results in terms of FNMR and FMR.

#### 3.3.3. Texture Based Methods

Monika and Kumar [25] propose the use of Local Binary Pattern (LBP) features as validation of minutiae matching. The images were pre-processed by binarization and skeletonization and the minutiae were extracted and matched using other authors’ techniques. On matched minutiae, LBP features in the neighborhood are computed and these features are also matched to validate the minutiae match. A subset of 20 images from the FVC2002 database was used. In both cases, the FNMR was 0%, with an FMR of 25% without the use of LBP validation, and an FMR of 20% with the proposed methodology, which resulted in an improvement.

Bakheet et al. [26] suggest a method based on the fusion of Speed-Up Robust Features (SURF) and Harris key points. Initially, the images were pre-processed through histogram equalization, normalization, segmentation, application of Gabor filters, and binarization. Then, Harris key points were extracted and SURFT descriptors in their neighborhood were extracted. Image matching is performed using the Euclidean distance between the descriptor vectors, using the RANSAC algorithm to refine the results and remove false matches. The FVC2000 DB1 and FVC2002 DB1 databases were used, achieving an accuracy of 92.5% and 95%, respectively.

Oriented Fast Rotated Brief (ORB) features are computed 100 times faster than SIFT features and 10 times faster than SURF features. Li and Shi [27] propose a methodology that compares a part of the fingerprint with the whole. To do this, the first step is to define a region of interest through the variance of the gray intensity. The ORB descriptors are then extracted and the best match is achieved by brute force using the hamming distance. Finally, the validation of the descriptor matching is done through the analysis of the second closest descriptor, the first being validated if the second presents a much greater distance. The FVC2004 database was used, and changes were made to the images to separate parts of the fingerprints. The proposed method achieved EER 2.83% (in 11 s) and was compared to a similar SIFT-based method that achieved 9.29% (in 123 s).

#### 3.3.4. Summary

Fingerprint recognition and comparison has been studied for more than 40 years, but the design of an accurate and interoperable system that requires little computational power is still considered an open problem.

Minutiae based methods are the most intuitive in terms of what is expected by human interpretation of fingerprint matching, and there are some considerations to be made: the models created must not be invertible for security reasons (not allow reconstruction of the fingerprint). The results achieved on the noisiest images (FVC2004) verify that all methods are very sensitive to the quality of the image since they are highly dependent on the minutiae extracted. The features extracted to describe the minutiae and build a model are of extreme importance. The proposed methodologies are based on the creation of a model that represents the fingerprint, with no methods proposed that analyse minutia by minutia, approaching the work that is performed manually in the forensic area.

The use of deep learning for fingerprint matching has some drawbacks. Firstly, there is the need to train a specific model for each situation, making the use of generic models unfeasible, which requires not only time, but also the acquisition of many images necessary for the training stage. Secondly, databases are often not balanced, with more comparisons of images to reject than images to match, so an imbalance in the data could lead to a biased model. Finally, there is the difficulty of generalizing the model to different sensors and image conditions, such as variations in brightness, for example, making practical applicability difficult.

The SIFT, SURF, ORB, and LBP features are techniques generally applied to image processing and object recognition. However, when the problem deals with fingerprint matching, there are some disadvantages such as the computing of the key points, which can be computationally intensive, introducing restrictions for real-time systems. These techniques extract features from the image, which may be advantageous when the minutiae are not well defined, but when compared to systems based on minutiae, these techniques present worse performance, being more often used as a complement to other algorithms.

## 4. Proposed Methodology

**Figure 7:**
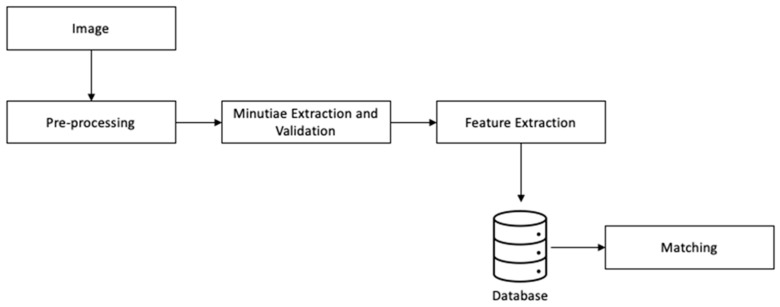
Proposed methodology.

This section describes the methodology implemented to process and search for matches between fingerprint images. The methodology is, in a larger picture, divided into four blocks (see Figure 7) which follow the common process. The new proposed methods are inserted into the pipeline.

The first block concerns image pre-processing in which the orientation and frequency of the ridges are estimated to apply oriented Gabor filters. Then, minutiae are extracted using the Crossing Numbers method and validated by defining a region of valid minutiae, removing nearby minutiae by type, and removing minutiae clouds. The third step concerns the creation of a set of polygons that represent each minutia, and finally, the last step is the minutiae matching and, subsequently, fingerprint match.

Considering the main goals of our study, which consist of proposing a methodology that can be used in real time, on a portable device, in forensic areas. We have to keep in mind that it is important to have a low computational effort such that any common hardware can run the algorithm and to provide a method that can be validated and understood by an operator. This said, we cannot use deep learning since we do not want to train a model for every scenario and there is a small amount of images. The texture based methods, in general, improve the results achieved by other methods but increase the computational effort and, therefore, we will not be using any for now. Each minutiae based method proposed in the literature uses the set of extracted (and sometimes filtered) minutiae to create a model for the fingerprint which is further compared to find a match. We will be focusing on validating each minutia individually which is the work that is carried out manually in real forensic scenarios.

### 4.1. Pre-Processing

Image pre-processing was implemented through the algorithm proposed by Raymond Thai [28] which includes the state of the art pre-processing techniques: segmentation, normalization, ridge orientation and frequency estimation, the application of oriented Gabor filters and skeletonization.

**Figure 8:**
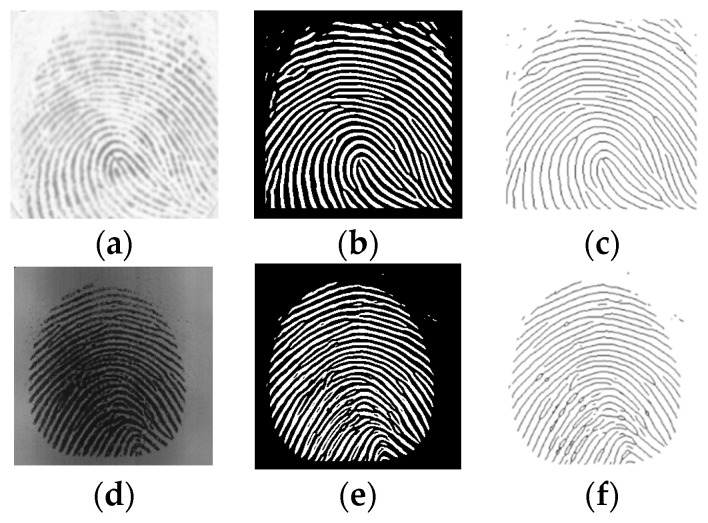
Image pre-processing: (**a**,**b**) are Original, (**c**,**d**) are Pre-processed, (**e**,**f**) are Skeletonized.

Figure 8 shows the effect of the applied pre-processing techniques in two images of different databases (FVC2000 DB1 and FVC2002 DB1).

### 4.2. Minutiae Extraction and Validation

Minutiae are extracted using the Crossing Numbers (CN) method in the skeletonized image. Due to noise in the image and damage in the fingerprints (ex: scars) there are some spurious minutiae that are captured by the CN method.

**Figure 9:**
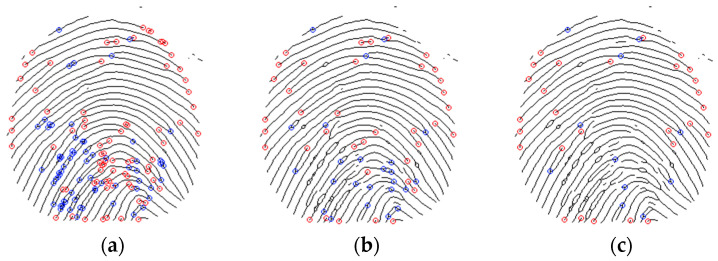
Minimum distance for minutiae by type: (**a**) δ=5 (**b**) δ=10 (**c**) δ=15.

Three techniques are implemented to create a set of valid minutiae from the extracted ones. The first step is to set a minimum distance δ between minutiae of the same type. Figure 9 represents the effect on the δ parameter in the excluded minutiae, having bifurcations in blue color and terminations in red.

**Figure 10:**
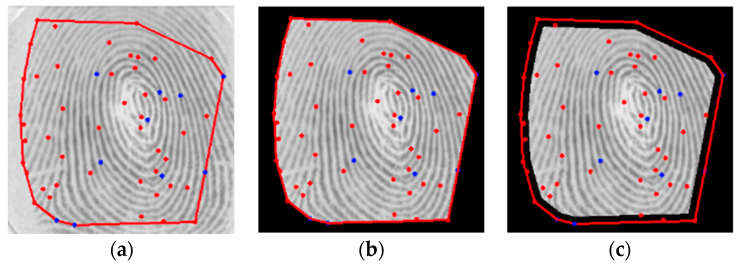
ROI definition: (**a**) Convex hull, (**b**) Convex border limits, (**c**) Erosion of γ=10 pixels (red represents a termination while blue represents a bifurcation minutia).

The second step is a novel definition of a region of interest (for valid minutiae) which is defined by the use of the convex hull. The convex hull was implemented using the incremental algorithm, and it defines the smallest convex polygon that contains all minutiae points in the set. Once the polygon is defined, an erosion of γ pixels is obtained through its interior (see Figure 10).

**Figure 11:**
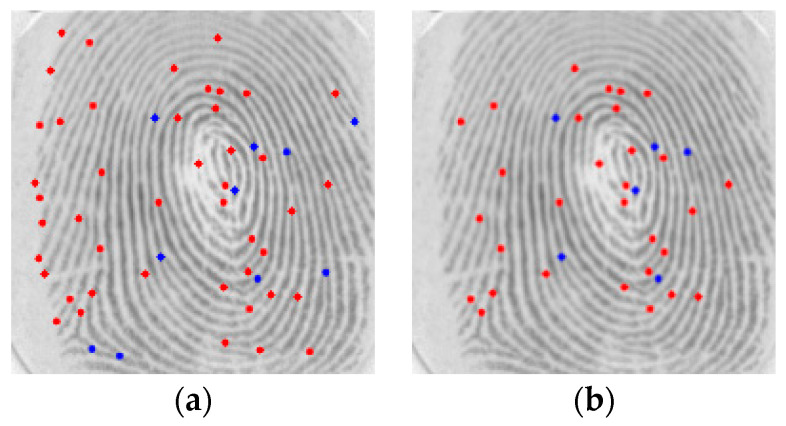
Set of minutiae: (**a**) Original (**b**) After ROI definition (red represents a termination while blue represents a bifurcation minutia).

This procedure excludes the border minutiae, which are not reliable for matching procedures, as shown in Figure 11.

**Figure 12:**
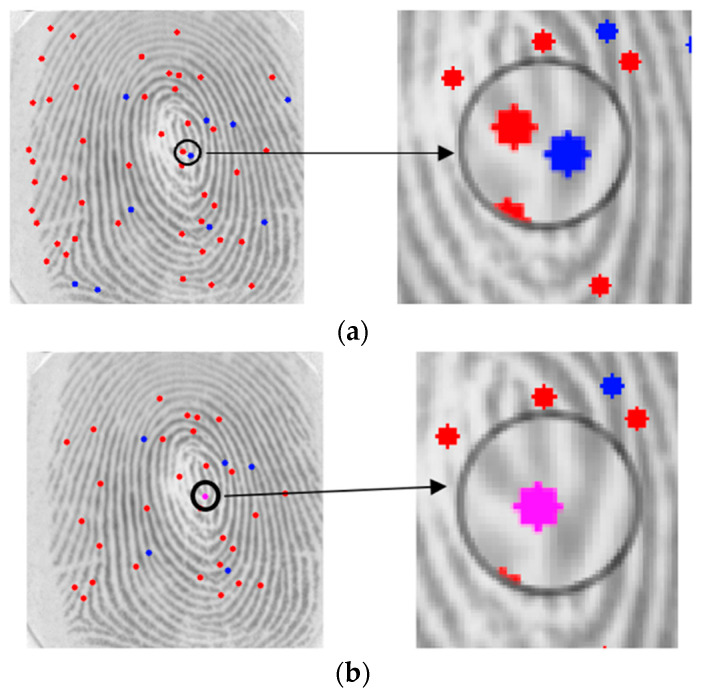
Minutiae Cloud Removal: (**a**) Original (**b**) Replaced (red represents a termination minutia, blue represents a bifurcation minutia, pink represents the new averaged minutia, the arrow and black circle emphasize the zoom).

The last step in minutiae validation is the removal of minutiae clouds, that is, when the algorithm finds a termination close to a bifurcation due to noise but only one is a real minutia (Figure 12). Minutiae clouds are replaced by the average point, creating a new averaged minutia.

### 4.3. Feature Extraction

Each valid minutia is individually represented through a set of polygons that are built using the neighboring minutiae as vertices. Then, each polygon is stored using its features, namely the edge size and the angles formed by adjacent vertices and the reference minutia. This means that each minutia will be associated with a set of fixed-length vectors that describe its associated polygons.

The first step in building the model to represent each minutia is to define n as is the number of desired vertices for each polygon. Then, a circumference centered on the reference minutiae is considered and its radius is incremented until the maximum radius is reached or a set of valid neighbor minutiae is found. The goal is to find at least n+2 minutiae and guarantee that their distribution matches at least one per quadrant. To increase the number of minutiae registered through polygons, sets of n points were also allowed but only if they were found when the maximum radius for the circumference is achieved.

**Figure 13:**
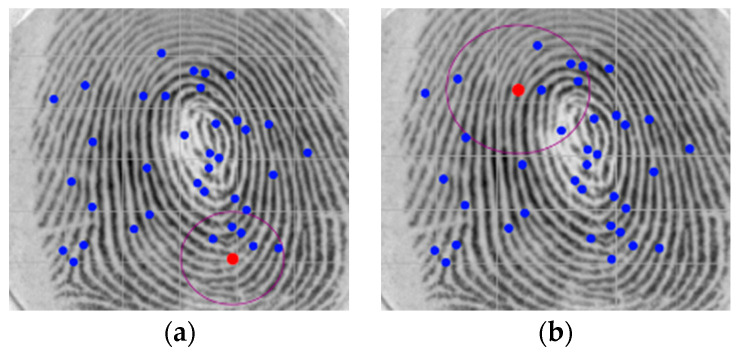
Search for neighbor minutiae: (**a**) Invalid; (**b**) Valid (blue represents a detected minutiae, purple circle represents the search radius for neighbor minutiae, red represented the reference minutia).

Figure 13 illustrates the search for neighbor minutiae to form the set of polygons that represents a minutia. After these minutiae are found, polygons with desired *n* vertices are formed using the points combinations. A polygon is valid if:

- There are no overlapping edges.
- There are no holes within the boundaries of the polygon.
- The starting point and the ending point coincide (closed polygon).
- The polygon contains the reference minutia.

**Figure 14:**
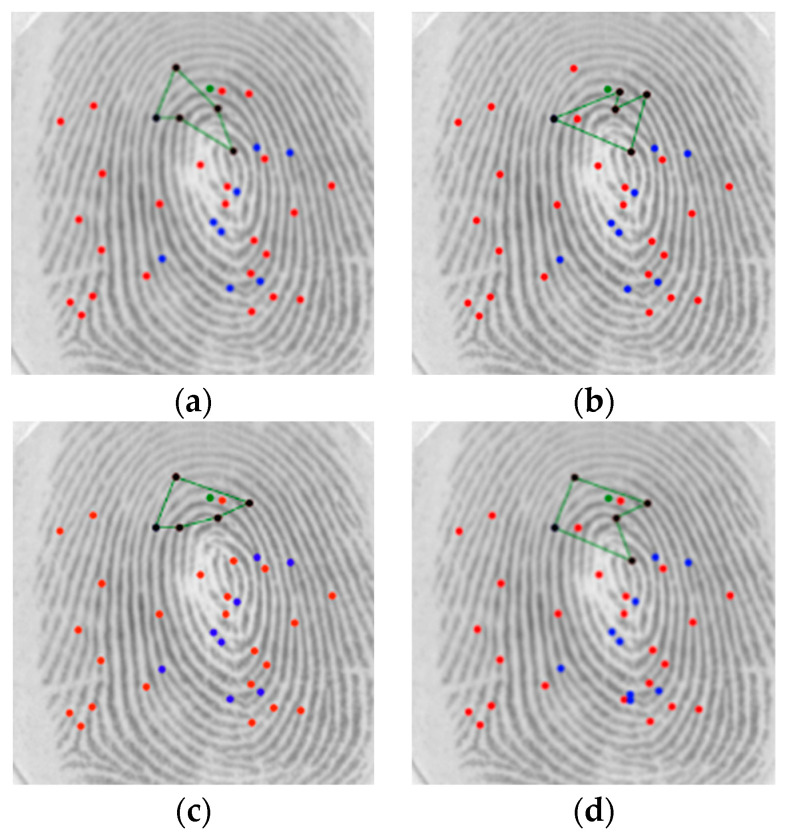
The green dot is the reference minutia; invalid polygons: (**a**,**b**); valid polygons: (**c**,**d**). Blue represents the termination minutia, red represents the bifurcation minutia, green dots represents the reference minutia, black represents the polygon vertices, green lines represents the polygon edges.

Figure 14 shows examples of both invalid and valid polygons that represent a minutia (reference is green and polygon vertices are black).

**Figure 15:**
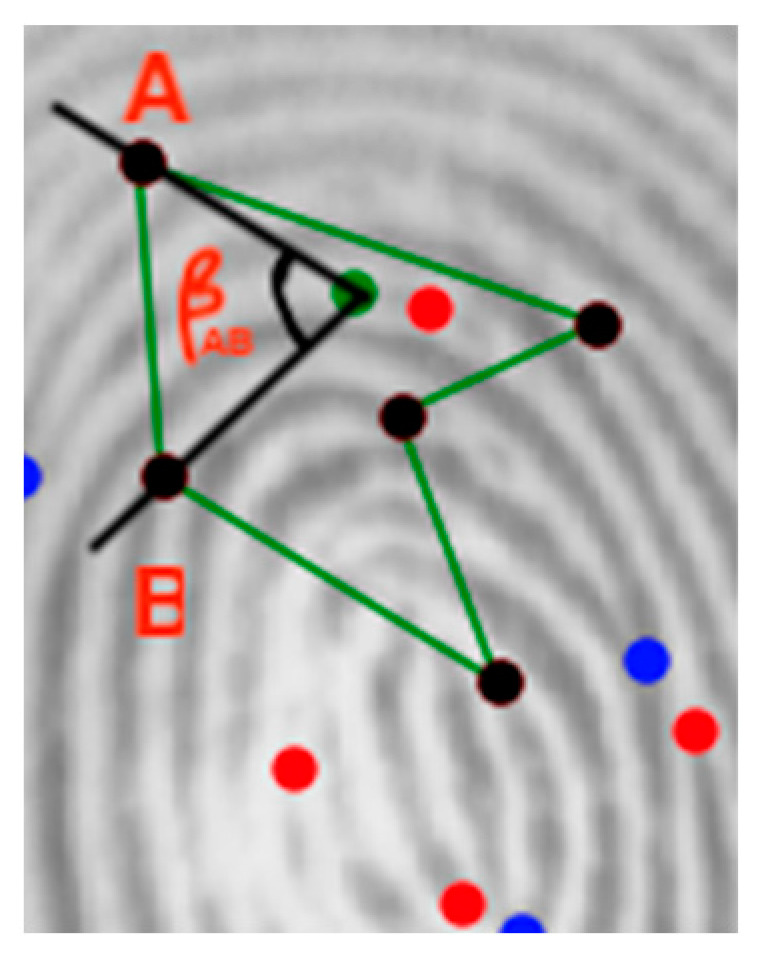
Angle used to build the feature vector (blue represents the termination minutia, red represents the bifurcation minutia, green dot represents the reference minutia, black represents the polygon vertices, green lines represents the polygon edges, A and B are names of the vertices).

A fixed-size feature vector is then built to represent each polygon using the sizes of the edges and the angles formed by the adjacent vertices and the reference minutia (see Figure 15).

**Figure 16:**
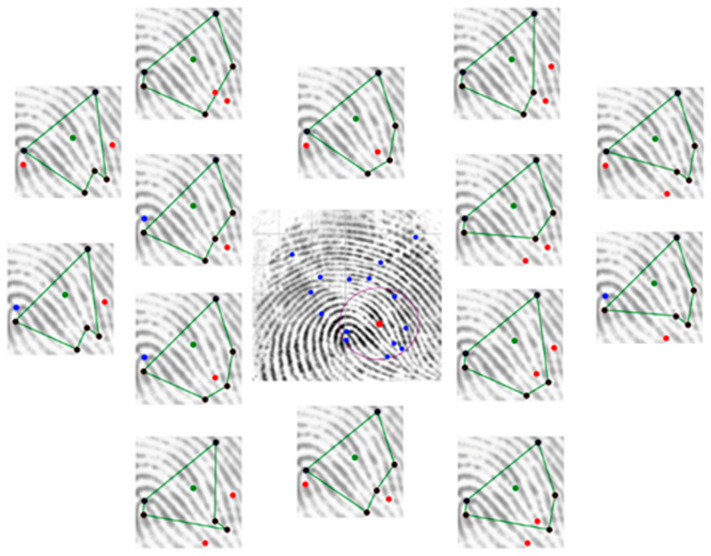
Set of valid polygons for a reference minutia (blue represents the termination minutia, red represents the bifurcation minutia, green dot represents the reference minutia, black represents the polygon vertices, green lines represents the polygon edges, purple circle represents the search radius).

Figure 16 is an example of all valid polygons that represent a valid reference minutia. Each polygon feature will be further considered for matching.

In this step, each minutia that was previously validated will be associated with a certain number of polygons that depend on its neighborhood minutiae. Each polygon is represented using a fixed-size vector that is not dependent on translations and rotations since it is always sorted in the same way (by keeping the larger edge size as most-left element and then keep the relative element order, and sorting the angles accordingly).

### 4.4. Matching

The matching has two steps: the minutiae matching and the fingerprint matching.

Minutiae matching is done through the polygons. Considering two minutiae, the set of polygons that represent each one is compared to find the closest match using the feature vectors. The best match is defined as the pair of feature vectors that has a smaller Euclidean distance. The pair that has smaller distance is not guaranteed to match (in totally different minutiae, there will always be a pair with smaller distance although it is a high distance compared to real matching minutiae). Thus, the pair of best matched polygons must be validated. The validation is performed using both an absolute and relative error criteria that is applied element-wise to the feature vectors. The thresholds applied to the edge sizes and the angles are different.

Fingerprint matching is conducted by defining a threshold TMC (Total Minutiae Corresponding) which is the minimum number of matching minutiae to consider the fingerprints as match. 

## 5. Results and Discussion

A random set of 125 images from FVC2000 DB1 database [11] was chosen which resulted in 125 genuine comparisons and 7154 imposter matchings. An average of 23 minutiae (represented by polygons) were registered.

The first experiments were performed to understand the importance of the values chosen for the relative and absolute error criteria that are used to validate the best match polygons. To start with some configuration, the number of vertices was set to n=5, the absolute error threshold for the edges size was set to ${TH}_{L}=5,$ and the absolute error threshold for the angles was set to ${TH}_{A}=10$. The threshold for fingerprint match was set as TMC=12 and the relative error threshold ${TH}_{REL}$ was varied. The results achieved are in Table 6.

The smaller the relative error threshold, the more polygons are validated using the absolute error threshold. This results in a more conservative system, which produces a higher number of false negatives, thus increasing the FNMR.

Then, the relative error threshold was fixed to ${TH}_{REL}=11$, and the other parameters were kept while varying ${TH}_{L}$. The following results were achieved (Table 7):

A smaller value of ${TH}_{L}$ means that it is harder for two polygons to match, and the system is conservative. The same absolute distance between two edge sizes produces a higher relative error if the edges are smaller. Thus, the absolute error criteria for edge sizes will have more importance to filter small edges that fail the relative error criteria.

Setting ${TH}_{L}=5$ and varying the absolute error threshold for the angles, the following results were achieved.

The results shown in Table 8 are important to understand that most of the angle verifications are done using the relative error criteria.

Using the best configuration found (${TH}_{REL}=11$, ${TH}_{L}=5$ and ${TH}_{A}=10$) the number of polygon vertices was changed.

Analyzing Table 9 we can state that, on one hand, the increase in the number of polygon vertices guarantees polygons with more distinct geometries and, as such, when a minutia is validated, the degree of confidence is greater, resulting in a decrease in the FMR since minutiae that do not hardly correspond are not matched. On the other hand, when increasing the number of polygon vertices, the algorithm becomes more dependent on the number of minutiae extracted, being more exposed to the quality of the database.

Varying the threshold *TMC* allows us to understand how good is the border between the number of minutiae that are correctly and wrongly matched, and compute the EER.

**Figure 17:**
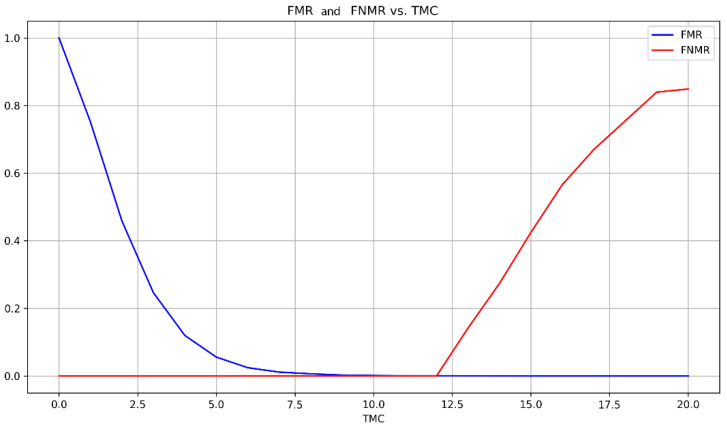
FMR and FNMR accordingly with *TMC* (FVC2000 DB1).

An EER of 0.03% was achieved at TMC=12. In general, the system behaves as expected for a fingerprint matching system, that is, a smaller threshold *TMC* allows more false images to match while a higher *TMC* reduces the false matches (Figure 17). 

**Figure 18:**
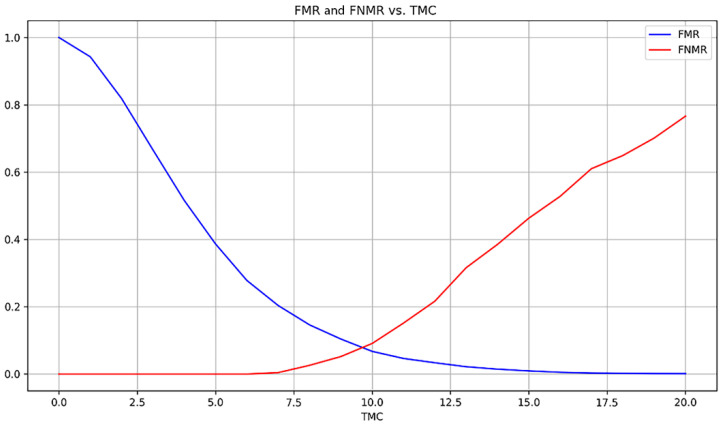
FMR and FNMR accordingly with *TMC* (FVC2002 DB1).

Keeping the algorithm parameters, more experiments were carried out using a random sample of 125 images from the FVC2002 DB1 database which led to an average of 35 registered minutiae (an increase of 52%). Initially, FNMR of 0% and FMR of 17.6% were achieved. These results suggest that the matching fingerprints have all more than 12 matching minutiae but 17.6% of the non-matching fingerprints are also reaching more than the *TMC* limit, which is expected since the high increase in the number of registered minutiae leads to hundreds of more possible polygons. Therefore, the error criteria were straightened and changed to ${TH}_{REL}=10$, ${TH}_{L}=4$ and ${TH}_{A}=10$. With the new configuration, an FNMR of 21.65% and an FMR of 3.36% were achieved, suggesting that the *TMC* threshold was not good for the images and, therefore, a swipe in the *TMC* values was done (see Figure 18).

An EER of 7.8% was achieved for a threshold TMC=9.5 but since the number of corresponding minutiae must be integer, using *TMC* = 10 FNMR of 9.10% and FMR of 6.74% were obtained.

A last set of experiments were carried out to challenge the algorithm and understand if it can deal with transformations applied to the images (such as translation and rotation) since the goal was to propose an algorithm that can be used in real-time without depending on the image alignment. From 15 random images chosen from FVC2000 DB1 database, a rotation of 45°, another rotation of 90° and a diagonal translation of 10% were applied, creating a new database composed by 60 images.

**Figure 19:**
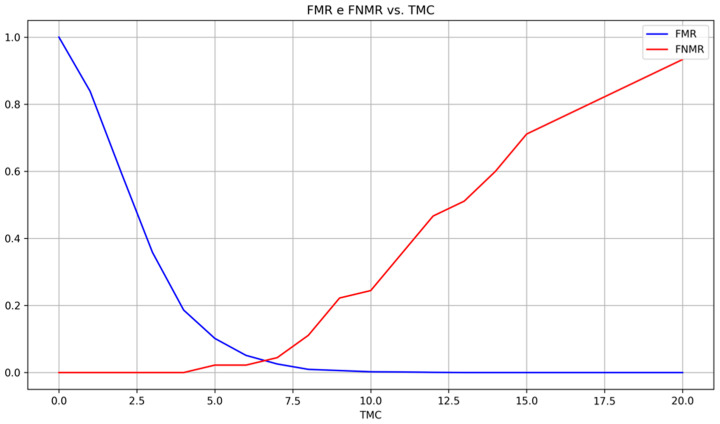
FMR and FNMR accordingly with *TMC* (transformed database).

With the previous configuration $n=5,\;{TH}_{REL}=11$, ${TH}_{L}=5,$ ${TH}_{A}=10$, and TMC=12, FNMR of 46.7% and FMR of 0.1% (Figure 19) were achieved meaning that false matchings are being well rejected but the number of matching minutiae is smaller even in matching images. Thus, those results suggest that the threshold *TMC* is too high.

Through the adjustment of *TMC* to 6.4, an EER of 3.6% was achieved, leading to an FNMR of 4.4% and an FMR of 2.6% using the integer threshold of 7, showing that the algorithm can identify matching minutiae without a previous image alignment. The results achieved show that the algorithm depends on the extracted and validated minutiae both in quality (to have the same geometrical relationship between minutiae in different matching images) and quantity (to ensure a higher number of registered minutiae), leading to the need of dynamically adjusting its parameters to achieve good results.

Compared to the state-of-the-art traditional methods, we can compare with the work from Mohamed-Abdul-Cader et al. [17], since they also choose a sample of around 150 images from FVC2002 DB1 and obtained EER of 6.68%. We achieved in 7.8% in a sample of the same database but we did not optimize the algorithm’s parameters in an exhaustive way. We overfitted the parameters in FVC2000 DB1 where we achieved 0.06% error and then performed only one iteration of tunning the parameters before attempt in FVC2002 DB1. This means that our results still have space for large improvement. Also, our results are in line with the state-of-the-art but our technique is a novelty since we are proposing a method that uses neighbor minutiae to validate the match of each individual minutia in opposition to what other authors do, using the set of minutiae as a whole.

Several authors have applied methods based on Deep Learning, such as He et al. [23] having achieved EER values between 3% and 6% (FVC2006 dataset) and between 7% and 9% in the AES3400 dataset. In the proposed method we achieved values of 7.8%, which are slightly higher than the values presented by He [23], justified by the fact that our initial objective was the development of a methodology able of running in an electronic device with low computational performance (example: cheap tablet) and capable of analyzing fingerprints in crime scenarios without internet access (that is, without access to the cloud).

## 6. Conclusions and Future Work

The design of a fingerprint matching system capable of acting on a large database is still an open problem and due to the unique characteristics of this biometric key, research continues to be carried out to propose more methods capable of handling fingerprints. This work aims at proposing a method that can act on a local database of fingerprints collected at a crime scene to carry out a selection of them and find those that are relevant to solving the crime in real time and without requiring a computational effort such that it can only be carried out on machines with high processing power and following the work that is done manually by forensic teams (this is, validating each minutia individually), so that the algorithm can be understood and validated by an operator.

In the state-of-the-art, methods were divided into three categories: methods based of deep learning, image texture, and minutiae. The first ones require high computational effort and the need to train models, which requires a high number of images and is not practical to be applied in real time. The second ones are based on image processing techniques that abstract themselves from the image and perform mathematical procedures and operation on the values of its pixels, being very sensitive to noise and presenting itself more as a validation complement of other methods. Finally, methods based on minutiae have the main disadvantage of depending on the reliability of minutiae extraction, but they are similar to the work carried out manually, allowing, for example, an operator to visualize and understand algorithm decisions and can be applied in real time without requiring a high computational effort (may be executed on portable devices).

The proposed methodology depends on the minutiae extracted from the image and aims to meet human reasoning, that is, to validate a minutia that is present in both images and check whether the number of validated minutiae reaches a level that allows us to guarantee that the fingerprints match. The algorithm is divided into four blocks: pre-processing, minutiae extraction and validation, feature extraction, and fingerprint matching. The first block was implemented with the application of the most mentioned state-of-the-art methods, resulting in images with substantial noise removal and clear distinction between ridges and valleys. In the second block, minutiae extraction was implemented using the Crossing Numbers method, which has proven results over the years, and minutiae validation was innovative, using the convex hull of the set of minutiae to define a region of valid minutiae. Feature extraction consists of the formation of polygons that represent a minutia, formed through neighboring minutiae. In the literature, most authors use minutiae triangulations to create models that represent the fingerprint, but with the aim of validating each minutia individually, the implement method associates each minutia with a set of unique polygons, with different shapes, depending on the arrangement, in space, of neighboring minutiae. Finally, fingerprint matching is intuitive; once a certain number of minutiae is associated in the two images, we consider the fingerprints to match.

The achieved results show that the implemented method is very dependent on the minutiae extracted, with different results and the need to make adaptations when changing databases. Image quality is a relevant factor, as it determines the details that are extracted. The concept of associating a set of polygons with a chosen number of vertices to each minutia proves to be challenging, since by increasing the number of polygon vertices we increase the certainty in the individual validation of each minutia, but more minutiae are required in the image and in the same relative position to ensure that there are enough of them to achieve an adequate number of minutia represented. Using dynamic parameters in the algorithm, namely the number of minutiae considered as a decision threshold to consider the fingerprints as match and the validation criteria of the polygon (maximum relative and absolute errors between the lengths of the edges and the angles formed between adjacent vertices and the reference minutia), FNMR 0% and FMR 0.06% were achieved in a random sample of 125 images from the FVC2000 DB1 database and FNMR 9.1% and FMR 6.7% in a random sample of 125 images from the FVC2002 DB1, results that are in line with the state of the art and suggest the possibility of transposing the method to real applications of the minutiae match. The application of the algorithm to images that underwent translation and rotation achieved FNMR 4.4% and FMR 2.6%, showing that the algorithm is capable of matching minutiae without requiring prior alignment of the images.

In future work, the dynamic adjustment of the polygon matching validation criteria would allow the automatic adaptation of the algorithm to different databases and the possibility of incorporating the use of minutiae that are valid but, because they are the extremes of the valid regions, they cannot be represented through polygons that surround them is proposed. Also, simple features of each polygon were chosen to allow the outlined objectives to be achieved, without increasing the computational effort but there is the possibility of exploring other features. 

## Figures and Tables

**Table 1:** Crossing number and type of minutia.

CN	Minutia
1	Termination
2	Middle ridge point
3	Bifurcation

**Table 2:** FVC2000 Database, adapted from [11].

FVC2000	Sensor	Image Size	Resolution
DB 1	Low-cost optical	300 × 300	500 dpi
DB 2	Low-cost capacitive	256 × 364	500 dpi
DB 3	Optical	448 × 478	500 dpi
DB 4	SFinGe	240 × 320	500 dpi

**Table 3:** FVC2002 Database, adapted from [12].

FVC2002	Sensor	Image Size	Resolution
DB 1	Optical	388 × 374	500 dpi
DB 2	Optical	296 × 560	569 dpi
DB 3	Capacitive	300 × 300	500 dpi
DB 4	SFinGe	288 × 384	500 dpi

**Table 4:** FVC2004 Database, adapted from [13].

FVC2004	Sensor	Image Size	Resolution
DB 1	Optical	640 × 480	500 dpi
DB 2	Optical	328 × 364	569 dpi
DB 3	Thermal sweeping	300 × 480	500 dpi
DB 4	SFinGe	288 × 384	500 dpi

**Table 5:** FVC2006 Database, adapted from [14].

FVC2006	Sensor	Image Size	Resolution
DB 1	Electric field	96 × 96	500 dpi
DB 2	Optical	400 × 560	569 dpi
DB 3	Thermal sweeping	400 × 500	500 dpi
DB 4	SFinGe	288 × 384	500 dpi

**Table 6:** Results as a function of the relative error criteria.

${TH}_{REL}\left(\%\right)$	Precision (%)	Recall (%)	F1 (%)	FNMR (%)	FMR (%)
1	100.0	75.5	86.0	24.5	0.00
3	100.0	75.5	86.0	24.5	0.00
5	100.0	78.0	87.8	21.7	0.00
7	100.0	82.1	90.2	17.9	0.00
9	98.1	95.3	96.7	4.7	0.03
11	96.4	100.0	98.2	0.0	0.06
13	94.6	100.0	97.3	0.0	0.08
15	82.2	100.0	90.2	0.0	0.32

**Table 7:** Results in function of the absolute error criteria for the edge sizes.

${TH}_{L}$	Precision (%)	Recall (%)	F1 (%)	FNMR (%)	FMR (%)
1	100.0	52.8	69.1	47.2	0.00
5	96.4	100.0	98.2	0.0	0.06
9	16.1	100.0	27.7	0.0	7.70
13	3.7	100.0	7.1	0.0	38.50

**Table 8:** Results as a function of the absolute error criteria for the angles.

${TH}_{A}$	Precision (%)	Recall (%)	F1 (%)	FNMR (%)	FMR (%)
5	100.0	68.9	81.6	31.1	0.00
10	96.4	100.0	98.2	0.0	0.06
15	96.4	100.0	98.2	0.0	0.06
20	96.4	100.0	98.2	0.0	0.06
30	95.5	100.0	91.7	0.0	0.07

**Table 9:** Results as a function of n.

n	Precision (%)	Recall (%)	F1 (%)	FNMR (%)	FMR (%)
3	2.9	99.1	5.7	0.9	48.80
4	29.3	92.5	44.4	7.6	3.31
5	96.4	100.0	98.2	0.0	0.06
6	100.0	75.5	86.0	24.5	0.00
7	100.0	67.9	80.9	32.1	0.00
8	100.0	58.5	73.8	41.5	0.00

## Data Availability

Data were obtained from FVC2000 database [11] and are publicly available upon request.
